# Against Alcohol

**Published:** 1891-10

**Authors:** 


### AGAINST ALCOHOL.

At the opening meeting of the recent international medical congress, which held its sessions at the national Prohibition camp ground in Port Richmond, S. I., Dr. N. S. Davis, of Chicago, spoke upon "The Nature and Effects of Alcoholic Liquor," in part as follows: "During 1890, 80,000,000 gallons of distilled spirits, 40,000,000 gallons of wine, 800,000,000 gallons of malt liquors, making a total of 920,000,000 gallons, were consumed in the United States. They cost the consumers $800,000,000, or $13 per head on the total population of the country.

The time lost from work, sickness and crimes due to drinking cost as much more, and it is estimated that rum cost the people of the United States in this period $1,600,000,000. What does the consumer get for this enormous expenditure? He does not get health, strength, clothing, food or happiness, but wasted fortunes, ruined lives and homes, homeless children, poor-houses, asylums and jails. Men who do not drink can work better and lose fewer days from sickness than those who do drink. People drink because of the erroneous ideas as to the nature and effects of alcoholic beverages. Before chemistry analyzed liquor it was suppsed to be stimulating, warming, soothing and restorative. This is now known to be fallacious. Nineteen-twentieths of the alcoholic drinks given to the sick are mixed with sugar, milk, eggs or meat broths, which furnish the nutriment and would support life better if given without alcohol. A five pound loaf of bread contains as much nutriment as will be found in a daily diet of eight or ten quarts of beer continued for a year. The man who drank this amount of beer would swallow a barrel of alcohol. In beer there is 4 per cent. of alcohol, in wine 15 per cent., and in distilled liquors from 50 to 60 per cent. Science classes alcohol as a poison. If taken pure it will destroy tissue as quickly as carbolic acid. Alcohol causes permanent structural changes in the liver, kidneys, stomach, heart, blood vessels and nervous tissue, and lessens the natural duration of life from ten to fifteen years.

Professor Williams H. Porter, of the New York Post Graduate School, followed with a paper on "Physiological Relation of Alcohol to Food," illustrated by charts showing the constituent parts of various articles of food and their value as nutritive agents. Starch, sugars and fats are stimulating and transformed into alcohol before they can be taken up by the system. Those used to eating food of this nature have a desire for stimulants.

Alcohol, being pleasant to the taste, is preferred to stimulating foods, and the alcoholic habit is gradually acquired. Albuminous food, such as meat, eggs and milk, stimulate the body without giving the desire for alcohol. The common practice of using vegetables and cereals to the exclusion of milk and meats often arouses a desire for alcoholic beverages. To get rid of the alcoholic habit, fats, starch and sugar should be avoided.

Professor Axel Gustafson told what he knew about "Some of the Effects of Alcohol on the Brain." He said that alcohol holds a preeminent place as a blood poison. This, he thinks, is shown in the crimes of alcoholists and drunkards. Suicide, insanity, idiocy and moral manias are traceable to the influence of alcohol on the brain.

